# Identification keys to the *Anopheles* mosquitoes of South America (Diptera: Culicidae). I. Introduction

**DOI:** 10.1186/s13071-020-04298-6

**Published:** 2020-11-18

**Authors:** Maria Anice Mureb Sallum, Ranulfo González Obando, Nancy Carrejo, Richard C. Wilkerson

**Affiliations:** 1 Departamento de Epidemiologia, Faculdade de Saúde Pública, Universidade de São Paulo, Avenida Doutor Arnaldo 715, São Paulo, São Paulo CEP01246-904 Brazil; 2 Departamento de Biología, Universidad del Valle, A.A 25360 Cali, Colombia; 3 Department of Entomology, Smithsonian Institution, National Museum of Natural History (NMNH), Washington, DC 20560 USA; 4 Walter Reed Biosystematics Unit, Smithsonian Institution Museum Support Center, 4210 Silver Hill Rd., Suitland, MD 20746 USA; 5 Walter Reed Army Institute of Research, 503 Robert Grant Avenue, Silver Spring, MD 20910 USA

**Keywords:** South America, *Anopheles*, Identification keys, Introduction

## Abstract

**Background:**

The worldwide genus *Anopheles* Meigen, 1918 is the only genus containing species evolved as vectors of human and simian malaria. Morbidity and mortality caused by *Plasmodium* Marchiafava & Celli, 1885 is tremendous, which has made these parasites and their vectors the objects of intense research aimed at mosquito identification, malaria control and elimination. DNA tools make the identification of *Anopheles* species both easier and more difficult. Easier in that putative species can nearly always be separated based on DNA data; more difficult in that attaching a scientific name to a species is often problematic because morphological characters are often difficult to interpret or even see; and DNA technology might not be available and affordable. Added to this are the many species that are either not yet recognized or are similar to, or identical with, named species. The first step in solving *Anopheles* identification problem is to attach a morphology-based formal or informal name to a specimen. These names are hypotheses to be tested with further morphological observations and/or DNA evidence. The overarching objective is to be able to communicate about a given species under study. In South America, morphological identification which is the first step in the above process is often difficult because of lack of taxonomic expertise and/or inadequate identification keys, written for local fauna, containing the most consequential species, or obviously, do not include species described subsequent to key publication.

**Methods:**

Holotypes and paratypes and other specimens deposited in the Coleção Entomológica de Referência, Faculdade de Saúde Pública (FSP-USP), Museo de Entomología, Universidad del Valle (MUSENUV) and the US National Mosquito Collection, Smithsonian Institution (USNMC) were examined and employed to illustrate the identification keys for female, male and fourth-instar larvae of *Anopheles*.

**Results:**

We presented, in four concurrent parts, introduction and three keys to aid the identification of South American *Anopheles* based on the morphology of the larvae, male genitalia and adult females, with the former two keys fully illustrated.

**Conclusions:**

Taxonomic information and identification keys for species of the genus *Anopheles* are updated. The need for further morphology-based studies and description of new species are reinforced.
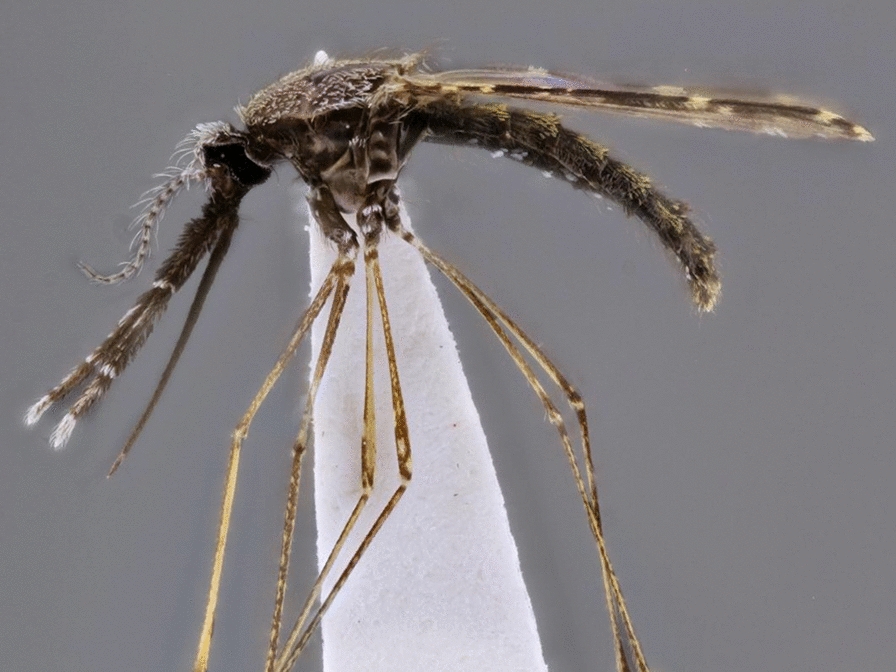

## Background

Malaria continues to be a serious public health problem. In 2016, there were an estimated 216 million cases of malaria in 91 countries, an increase of 5 million cases over 2015. Malaria deaths reached 445,000 in 2016, a similar number (446,000) to 2015 (http://www.who.int/news-room/fact-sheets/detail/malaria) [1]. In 2017, there were an estimated 219 million cases of malaria worldwide, mostly in developing countries, resulting in an estimated 435,000 deaths of which 266,000 deaths were children under 5 years of age [2].

In Central and South America, malaria transmission occurs primarily at altitudes of less than 1000 meters above sea level. It is not surprising therefore that the incidence of malaria is highest in lowland countries, such as those situated in the Amazon basin: Brazil, Colombia, Venezuela, Ecuador, Peru, and the Guianas. Brazil, Colombia, Peru, and Venezuela contributed approximately 82% of the 875,000 malaria cases reported in the region in 2016: Brazil (18.0%); Colombia (15.3%); Peru (14.3%) and Venezuela (34.4%) [1]. In 2017, an increase in malaria cases was reported in Brazil, Ecuador, Mexico, Nicaragua, and Venezuela [2].

Variability in the epidemiological components of malaria transmission (host(s), parasites, environment) varies widely by locality, with differences in physiography, regional and local ecological characteristics, vector competence and vectorial capacity of individual species of *Anopheles* Meigen, 1818. Mosquito vectors can differ in physiology, behavior, and ecology, as well as in morphological characters used for their identification. These differences taken together can facilitate the definition of species and are the source of characteristics for species identification. The most accessible method of identification is by morphological characteristics. However, if basic (*alpha*) morphological taxonomy is incomplete, it is possible to make incorrect identifications of morphologically similar species of very different public health importance. This is especially the case for species complexes that include both vector and non-vector species. The definition and delineation of morphologically similar species often requires auxiliary tools, for example molecular markers, which include DNA sequences. DNA not only has given us evolutionary insights but also many new characters for the recognition and identification of species. However, molecular methods are not available in many malaria-endemic areas in South America. Recently, multiple studies (e.g. [3–9]) have reported satisfactory resolution of taxonomic problems using a combination of molecular markers, and/or morphological characters from all developmental stages. These studies have led to description or re-description of multiple species, and the recognition of complexes of morphologically similar species, which are summarized here.

In spite of technological and analytical advances, the identification of mosquito species using external morphological characters is still a preferred method since microscopes are easy to use, relatively inexpensive and can also be employed to study live specimens for ecological studies, such as capture-mark-release-recapture. Even though there are many keys addressing morphological identification of Neotropical *Anopheles*, they are limited in geographical or taxonomic scope and/or are outdated. For this reason, we present comprehensive keys (based on the morphology of male genitalia, females, and fourth-instar larvae) to all *Anopheles* species recorded in South America.

Mosquitoes (family Culicidae Meigen, 1818), belong to order Diptera (true flies, i.e. insects with two wings), and like many other insect orders (Coleoptera, Hymenoptera, etc.) they are holometabolous, meaning they have distinctly different egg, larval, pupal and adult stages. The culicids are recognized as a monophyletic group (with a single common ancestor) [10–12], that diverged from its nearest relative, family Chaoboridae Edwards, 1912, about 255 million years ago (mya) [13]. The two culicid subfamilies, Anophelinae Grassi, 1900 and Culicinae Meigen, 1818, diverged 229–192 mya [14].

**Fig. 1:**
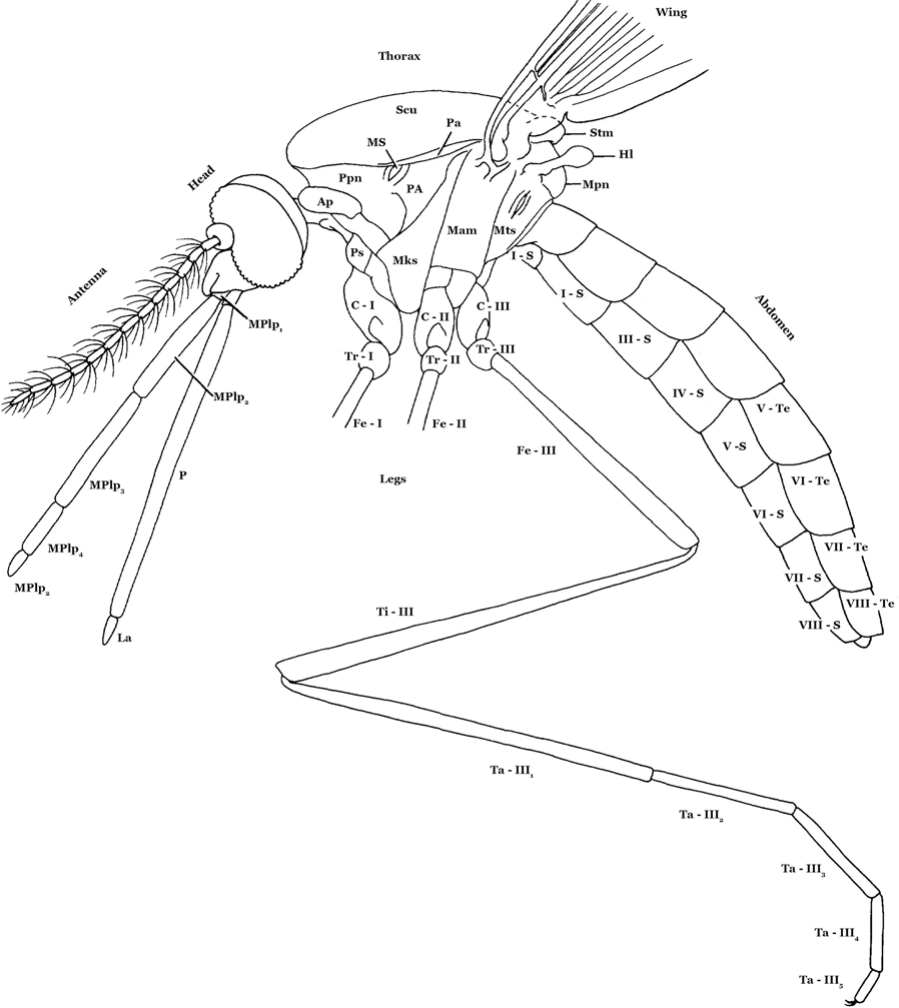
Female anopheline mosquito, lateral view. *Abbreviations*: Ap, antepronotum; C-I, forecoxa; C-II, midcoxa; C-III, hindcoxa; Fe-I, forefemur; Fe-II, midfemur; Fe-III, hindfemur; Hl, halter; La, labellum; Mks, mesokatepisternum; Mm, mesepimeron; MPlp_1–5_, maxillary palpus, segments 1–5; Mpn, mesopostnotum; MS, mesothoracic spiracle; Mts, metepisternum; P, proboscis; Pa, paratergite; PA, postspiracular area; Ppn, postpronotum; Ps, proepisternum; S-I-VIII, sterna I-VIII; Scu, scutum; Stm, scutellum; Ta-III_1–5_, hindtarsomeres 1–5; Te-I-VIII, terga I-VIII; Ti-III, hindtibia; Tr-I, foretrochanter; Tr-II, midtrochanter; Tr-III, hindtrochanter

Adult mosquitoes (Fig. 1) can be distinguished [15] by: scales on wing veins and usually also on the head, legs, thorax and abdomen; proboscis long, extending well beyond the clypeus; characteristic wing venation (also found, however, in families Dixidae Schiffner, 1868 and Chaoboridae) [16] (i.e. subcostal (Sc) vein ending near or beyond midpoint of the costal vein (C), Sc and vein R_1_ both reach C in the apical half of the wing in front of R_2_ and R_3_, and vein M three-branched); antennal pedicel prominent and nearly always larger than the scape. Mosquito (and chaoborid) larvae [16, 17] have the three thoracic segments fused into a rounded composite structure that is wider than the head or abdomen; abdominal segment X with a fan-like ventral brush; lateral tufts of long setae on most thoracic and abdominal segments; labrum with a distinctive brush of long setae on either side (Culicidae only); and antenna moderately long, usually with a number of apical setae (Culicidae only).

While the above characters serve for identification, another overlapping set of characters further characterize the family and can be used for phylogenetic analyses [18]. According to [10], the synapomorphies of Culicidae are internal premandibles in the larvae, apparently without residual external sclerites, and a long proboscis, with stilettes corresponding to the maxillae, mandibles and the labrum-hypopharynx, all encased in the labium [16]. In a study using characters of the adults and fourth-instar larvae, Harbach et al. [12] defined three synapomorphies for adult Culicidae: presence of erect scales on the head, dorsally; mouthparts forming a long proboscis; and the presence of prealar setae. In the larval stage, mosquitoes can be identified by: a well-sclerotized head that is clearly separated from the thorax; legless thoracic segments that are little differentiated from each other; and an abdomen made up of 10 segments, but with only nine apparent. The larvae are metapneustic (single spiracle in the post-abdominal region) with a spiracular plate that opens at the end of a siphon or on a small dorsal lobe that has a narrow, sclerotized posterior band in the dorsal region of abdominal segment VIII [12], and frequently with two pairs of anal papillae inserted at the end of segment X.

The Culicidae, and related families (Dixidae, Chaoboridae and Corethrellidae Edwards, 1932), pass through four larval instars (first-, second-, third- and fourth-instar) before developing into the pupal stage. The pupal and larval stages have serially (segmentally) homologous setae that have also been shown to be homologous between the larval and pupal stages (e.g. [19–21]). A common numbering system for these setae makes comparison of taxa straightforward.

Currently, the family Culicidae includes nearly 3600 valid species and a number of subspecies, which would add up well over 3600 [22] within two subfamilies (Anophelinae and Culicinae). Subfamily Anophelinae (genera *Anopheles*, *Chagasia* Cruz, 1906 and *Bironella* Theobald, 1905) contains about 500 described species. Most species of the Anophelinae can be distinguished in the adult stage from those of the subfamily Culicinae by the female maxillary palpus about as long as the proboscis, the male palpus also long and usually clubbed apically, a rounded (not tri-lobed) scutellum (except genus *Chagasia*), and the characteristic elevated angle of the abdomen at rest or when feeding. The larvae have no respiratory siphon and characteristically lie parallel to the water surface, although some other mosquitoes, such as genus *Uranotaenia* Lynch Arribálzaga, 1891, have a very short siphon and can be mistaken for *Anopheles* at first glance. The larvae of most species of *Anopheles* have at least some abdominal seta 1 broadened and leaf-like, which allows the larva to take advantage of surface tension to remain parallel to the surface.

The internal classification and phylogeny of the genus *Anopheles* has been reported by Harbach & Kitching [23], Harbach [24], Sallum et al. [25, 26], Foster et al. (2017) [27]. Worldwide there are three genera in the subfamily Anophelinae; *Anopheles*, *Bironella* and *Chagasia* (but see [27]). Most species belong to genus *Anopheles* while *Bironella* (Australasian) and *Chagasia* (Neotropical) have eight and five species respectively, which are not of medical importance. Genus *Anopheles* has seven subgenera (but see [27] and [28]): *Anopheles* (183 species); *Baimaia* Harbach, Rattanarithikul & Harrison, 2005 (1 species); *Cellia* Theobald, 1902 (224 species); *Christya* Theobald, 1903 (2 species); *Kerteszia* Theobald, 1905 (12 species); *Lophopodomyia* Antunes, 1937 (6 species); *Nyssorhynchus* Blanchard, 1902 (40 species); *Stethomyia* Theobald, 1902 (5 species) (Table 1), and approximately 13 recently discovered new species of the subgenera *Anopheles* and *Nyssorhynchus* [29]. Species of all subgenera except for *Baimaia* and *Cellia* occur in South America, and species of the subgenera *Kerteszia*, *Lophopodomyia*, *Nyssorhynchus* and *Stethomyia* are only found in the Neotropics. The largest cosmopolitan genera are *Anopheles* and *Cellia*. From a malaria transmission standpoint, a relatively small number of species of subgenus *Cellia* (i.e. the Gambiae Complex) are responsible for much of world’s malaria transmission [8]. The subgenera are further subdivided into informal morphologically similar groupings, usually for convenience, but which often lack phylogenetic significance [24].

**Table 1:** Valid species of the genus *Anopheles* of the subgenera *Anopheles*, *Kerteszia*, *Lophopodomyia*, and *Stethomyia* found in South America, grouped by subgenus and series

Subgenus/Series	Species, authorship, date
*Anopheles* Meigen, 1818 Series Arribalzagia	*anchietai* Corrêa & Ramalho, 1968^a^
	*annulipalpis* Lynch Arribálzaga, 1878
	*apicimacula* Dyar & Knab, 1906
	*bustamantei* Galvão, 1955
	*calderoni* Wilkerson, 1991^b^
	*costai* da Fonseca & da Silva Ramos, 1940^a^
	*evandroi* da Costa Lima, 1937
	*fluminensis* Root, 1927^a^
	*forattinii* Wilkerson & Sallum, 1999^a^
	*guarao* Anduze & Capdevielle, 1949
	*maculipes* (Theobald, 1903)^a^
	*malefactor* Dyar & Knab, 1907
	*mattogrossensis* Lutz & Neiva, 1911^a^
	*medialis* Harbach, 2018 (new name for *An*. *intermedius* Chagas, 1908)^a^
	*mediopunctatus* (Lutz, 1903)^a^
	*minor* da Costa Lima, 1929^a^
	*neomaculipalpus* Curry, 1931^b^
	*peryassui* Dyar & Knab, 1908^a^
	*pseudomaculipes* (Chagas in Peryassú, 1908)
	*punctimacula* Dyar & Knab, 1906
	*rachoui* Galvão, 1952
	*shannoni* Davis, 1931
	*vestitipennis* Dyar & Knab, 1906
Series Anopheles	*eiseni eiseni* Coquillett, 1902
	*eiseni geometricus* Corrêa, 1944^a^
	*pseudopunctipennis levicastilloi* Levi Castillo, 1944
	*pseudopunctipennis neghmei* Mann, 1950
	*pseudopunctipennis noei* Mann, 1950
	*pseudopunctipennis patersoni* Alvarado & Heredia, 1947
	*pseudopunctipennis pseudopunctipennis* Theobald, 1901^b^
	*pseudopunctipennis rivadeneirai* Levi Castillo, 1945
	*tibiamaculatus* (Neiva, 1906)^a^
*Kerteszia* Theobald, 1905	*auyantepuiensis* Harbach & Navarro, 1996
	*bambusicolus* Komp, 1937^a^
	*bellator* Dyar & Knab, 1906^a^
	*boliviensis* (Theobald, 1905)
	*cruzii* Dyar & Knab, 1908^a^
	*gonzalezrinconesi* Cova-García, Pulido F. & Escalante de Ugueto, 1977
	*homunculus* Komp, 1937^a^
	*laneanus* Corrêa & Cerqueira, 1944^a^
	*lepidotus* Zavortink, 1973
	*neivai* Howard, Dyar & Knab, 1913^b^
	*pholidotus* Zavortink, 1973^b^
	*rollai* Cova-García, Pulido F. & Escalante de Ugueto, 1977^a^
*Lophopodomyia* Antunes, 1937	*gilesi* (Neiva in Peryassú, 1908)^a^
	*gomezdelatorrei* Leví-Castillo, 1955^a^
	*oiketorakras* Osorno-Mesa, 1947^a^
	*pseudotibiamaculatus* Galvão & Barretto, 1941^a^
	*squamifemur* Antunes, 1937^a^
	*vargasi* Gabaldón, Cova García & López, 1941^a^
*Stethomyia* Theobald, 1902	*acanthotorynus* Komp, 1937^a^
	*canorii* Floch & Abonnenc, 1945
	*kompi* Edwards, 1930^a^
	*nimbus* (Theobald, 1902)^a^
	*thomasi* Shannon, 1933^a^

*Note*: Sources of specimens used to photograph fourth-instar larvae and male genitalia are denoted as: ^a^ FSP-USP, Brazil; ^b^ MUSNUVE, Colombia

Recently, Foster et al. [27] proposed that the Neotropical subgenera of *Anopheles* should be elevated to genus rank, see [28] for discussion of these changes, which were proposed subsequent to the writing of our keys. If these subgenera are considered by future authors to be genera, agreement of genus-species gender and inclusion or deletion of parentheses around author names as appropriate will be needed. For example, *An*. (*Ker*.) *boliviensis* (Theobald, 1905) would be *Kerteszia boliviensis* Theobald since it was originally described in genus *Kerteszia*.

In South America, the genus *Anopheles* has approximately 86 formally named species, with many yet to be named. Some *Anopheles* are associated with undisturbed forested areas while others are more abundant in areas that have been severely altered ecologically as a result of human activities, such as farming and logging. In the Neotropics, the genus *Anopheles* consists mostly of subgenera not found elsewhere in the world (see Table 1 for species, authors, and publication dates). Even the widespread subgenus *Anopheles* is comprised mostly of a group of species unique to the region (Series Arribalzagia). Phylogenetic studies have shown that the genus *Chagasia*, the earliest extant branch in the subfamily Anophelinae, is found only in Latin America. This has led to the quite plausible assertion that genus *Anopheles* originally evolved in this region of the world [24].

### Subgenus *Anopheles*

Subgenus *Anopheles* (with 183 species) is represented by 29 species in the Neotropics and an additional number of putative species, which were newly discovered by Bourke et al. [29]. Most belong to Arribalzagia Series (23 species), a group only found in Central and South America. Most of the species of the series are forest mosquitoes, found in swamps or slow-moving streams. *Anopheles* (*Anopheles*) *pseudopunctipennis* Theobald, 1901 (Anopheles Series, Pseudopunctipennis Group) is widespread, found in freshwater sometimes at higher elevations, usually in association with the green alga *Spyrogyra* Nees, 1820. Some species in this subgenus are important in malaria transmission, e.g. *An.* (*Anopheles*) *calderoni* Wilkerson, 1991, *An.* (*Anopheles*) *fluminensis* Root, 1927, *An.* (*Anopheles*) *pseudopunctipennis*, *An.* (*Anopheles*) *punctimacula* Dyar & Knab, 1906, and *An.* (*Anopheles*) *vestitipennis* Dyar & Knab, 1906.

### Subgenus *Kerteszia*

Larvae of the subgenus *Kerteszia* (12 species) almost exclusively utilize water collections in species of the mostly Neotropical plant family Bromeliaceae (bromeliads). They are most similar to larvae of the subgenus *Nyssorhynchus* but can be distinguished by a number of morphological characters [30], including differing setae on the gonocoxites [24]. Some species of *Kerteszia* have historically been quite important malaria vectors (e.g. *An. bellator* Dyar & Knab, 1906*, An*. *cruzii* Dyar & Knab, 1908*, An*. *homunculus* Komp, 1937, *An*. *neivai* Howard, Dyar & Knab, 1913, and *An*. *pholidotus* Zavortink, 1973). Logging activities diminish larval habitats, as many bromeliads are only found in trees. *Anopheles bambusicolus* Komp, 1937, as its name might imply, are the only *Kerteszia* spp. not to occupy bromeliads, but instead are found in unbroken bamboo internodes. One of us (RW) also found larvae in Amazonian nut pods in Peru (unpublished observation), and in a discarded tire in Brazil [31].

### Subgenus *Lophopodomyia*

Larvae of the subgenus *Lophopodomyia* (six species) are found in forested, shaded habitats in small slow-moving streams. They are similar to larvae of the subgenus *Anopheles*, but can be distinguished by characteristics of the male genitalia [24], long setae on the prothoracic and mesothoracic pleural groups (P,M-9-12), well-developed palmate setae, and fringed pupal paddles. Females are not known to be of medical importance but can blood-feed on humans when they enter the forest environment [32].

### Subgenus *Nyssorhynchus*

Larvae of the subgenus *Nyssorhynchus* (40 species with numerous cryptic unnamed species) are found in a large variety of open or partly shady areas [32]. Species included in the *Nyssorhynchus* are most closely related to species of the subgenus *Kerteszia* but can be distinguished by several morphological characters [30]. The subgenus contains many important malaria vectors including *An*. *albimanus* Wiedemann, 1820 (malaria vector in Central America and northern South America), species of the *An*. *albitarsis* Lynch Arribálzaga, 1878 complex (9 species), *An*. *aquasalis* Curry, 1932 (found in brackish waters), *An*. *darlingi* Root, 1926 (commonly associated with rivers and streams), *An*. *nuneztovari* Gabaldon, 1940, *An*. *triannulatus* (Neiva & Pinto, 1922), and many comprise species complexes.

### Subgenus *Stethomyia*

Larvae of the subgenus *Stethomyia* (5 species) are, as in *Lophopodomyia*, forest species found in well-shaded small streams and swampy areas. Adults are mostly dark-colored but have a characteristic silvery stripe on the scutum. The setae of the gonocoxite are distinctive [24]. In the larva, head seta 2-C are widely separated, reminiscent of subgenus *Cellia*, thoracic setae P,M,T-9–12 have thorn-like branches, and the abdomen lacks distinct palmate setae. The species are not known to be of medical importance, but females will bite humans [32].

### Distribution of Neotropical *Anopheles* spp.

The distributions of the species treated here are summarized in Table 2. Location should be considered as one of the most important “characters” used for identification. For example, if a species is only known from east of the Andes mountains but is reported from the Pacific side of the cordillera, it should be flagged as a possible misidentification or perhaps evidence of cryptic diversity. Likewise, species reported well outside known recorded ranges should be carefully evaluated. The combination of species found in a given country is usually unique since ecologies are heterogeneous among countries (or states, provinces, etc.). In fact, most identifiers will first ask the question “Where was it collected?” before selecting a key.

**Table 2:** Distributions of the *Anopheles* species, sorted by subgenus, based on data contained in the on-line catalog of the Walter Reed Biosystematics Unit (http://www.mosquitocatalog.org/) and more recent publications [65–81]

Species	AR	BO	BR	CL	CO	EC	GF	GY	PY	PE	SR	UY	VE
*Stethomyia*													
* acanthotorynus*							×			×			
* canorii*							×						
* kompi*			×		×		×			×	×		×
* nimbus*		×	×		×		×	×		×			×
* thomasi*			×		×					×			×
*Anopheles*													
* anchietai*	×		×										
* annulipalpis*	×											×	
* apicimacula* (*s.l.*)	×	×			×	×		×			×		×
* bustamantei*			×										
* calderoni*					×	×				×			×
* costai* (*s.l.*)		×	×		×		×			×	×		×
* eiseni eiseni*		×	×		×	×	×	×		×	×		×
* eiseni geometricus*			×										
* evandroi*	×		×										
* fluminensis* (*s.l.*)	×	×	×		×	×			×	×			×
* forattinii*			×		×	×	×			×			×
* guarao*													×
* maculipes*	×	×	×				×		×			×	
* malefactor*					×	×							×
* mattogrossensis*		×	×		×	×				×			×
* medialis*	×	×	×		×	×	×	×	×	×	×		×
* mediopunctatus*	×		×		×		×	×		×	×		
* minor*	×		×				×		×		×		
* neomaculipalpus*	×	×	×		×	×			×	×			×
* peryassui*		×	×		×		×	×		×	×		×
* pseudomaculipes*			×		×					×		×	
* pseudopunctipennis*	×	×	×	×	×	×	×	×	×	×	×		×
*levicastilloi*													
* p. neghmei*				×									
* p. noei*				×									
* p. patersoni*	×												
* p. pseudopunctipennis*	×	×	×	×	×	×	×	×	×	×	×		×
* p. rivadeneirai*						×							
* punctimacula*	×	×	×		×	×		×				×	×
* rachoui*			×										
* shannoni*		×	×		×	×		×		×	×		×
* tibiamaculatus*	×	×	×							×			
* vestitipennis*					×			×	×				
*Lophopodomyia*													
* gilesi*		×	×		×	×		×					×
* gomezdelatorrei*						×							
* oiketorakras*					×								
* pseudotibiamaculatus*			×										
* squamifemur*		×	×		×	×	×			×			×
* vargasi*													×
*Nyssorhynchus*													
* albertoi*			×										
* albimanus*			×		×	×				×	×		×
* albitarsis* (*s.s.*)	×	×	×		×				×			×	×
* albitarsis* F					×								×
* albitarsis* G			×										
* albitarsis* H			×										
* albitarsis* I			×		×								×
* antunesi*	×		×		×		×					×	
* aquasalis*	×		×		×	×	×	×	×		×	×	×
* argyritarsis*	×	×	×		×	×	×	×	×	×		×	×
* arthuri* (*s.s.*)			×										
* arthuri* B			×										
* arthuri* C			×										
* arthuri* D			×										
* atacamensis*				×									
* benarrochi*		×	×		×		×	×	×			×	×
* benarrochi* B					×					×			
* braziliensis*	×	×	×		×		×	×		×	×		×
* darlingi*	×	×	×		×	×	×	×	×	×	×	×	×
* deaneorum*	×	×	×						×				
* dunhami*			×		×					×			
* evansae* (*s.l.*)	×	×	×		×	×			×	×		×	×
* galvaoi*	×		×							×			
* goeldii*			×								×		
* guarani*	×		×										
* halophylus*			×										
* ininii*	×	×	×				×	×	×				
* janconnae*			×										×
* konderi* (*s.l.*)		×	×							×			
* lanei*			×										
* lutzii* (*s.l.*)	×	×	×						×	×			
* marajoara*		×	×										
* nigritarsis*	×		×										
* nuneztovari* (*s.l.*)		×	×		×	×	×	×		×	×		×
* oryzalimnetes*			×						×				
* oswaldoi* (*s.s.*)			×										
* oswaldoi* A			×		×								
* oswaldoi* B					×	×				×			
* oswaldoi* (*s.l.*)	×	×	×		×	×	×	×	×	×	×	×	×
* parvus*	×	×	×	×	×				×	×			×
* pictipennis*	×		×	×									
* pristinus*			×										
* rangeli*		×	×		×	×				×			×
* rondoni*	×	×	×						×				×
* sanctielii*							×						
* sawyeri*			×										
* striatus*			×										
* strodei* (*s.l.*)	×	×	×		×	×	×	×	×	×	×		×
* triannulatus* (*s.s.*)	×	×	×		×	×	×	×	×	×	×		×
* triannulatus* C			×										
* trinkae*		×			×	×				×			
*Kerteszia*													
* auyantepuiensis*													×
* bambusicolus*	×	×	×		×	×	×	×		×	×		×
* bellator* (*s.l.*)			×					×			×		×
* boliviensis*		×	×		×	×	×	×	×	×	×		×
* cruzii* (*s.l.*)	×	×	×		×	×	×	×		×	×		×
* gonzalerinconesi*													×
* homunculus* (*s.l.*)		×	×		×		×	×		×			×
* laneanus*	×	×	×							×			
* lepidotus*		×			×	×				×			
* neivai* (*s.l.*)		×	×		×	×	×	×		×	×		×
* pholidotus*		×			×	×				×			×
* rollai*													×

*Abbreviations*: AR, Argentina; BO, Bolivia; BR, Brazil; CL, Chile; CO, Colombia; EC, Ecuador; GF, French Guiana; GY, Guyana; PY, Paraguay; PE, Peru; SR, Suriname; UY, Uruguay; VE, Venezuela

## Methods

Illustrations for keys are photomicrographs or drawings. Photomicrographs were taken using a digital Canon Eos T3i, attached to a light Diaplan Leitz microscope, using the program Helicon Focus, which was used to build single in-focus images by stacking multiple images of the same structure. Photomicrographs of the male genitalia were taken from specimens from FSP-USP and MUSENUV. For few species, illustrations were reproduced from published literature. Keys are presented in Part II for the fourth-instar larvae [33], Part III for the male genitalia [34], and Part IV for the adult females [35].

## Results and discussion

As a basis for these keys, the primary types (holotypes and paratypes) and other specimens deposited in the Coleção Entomológica de Referência, Faculdade de Saúde Pública, Universidade de São Paulo, São Paulo, Brazil (FSP-USP) [36–38], Museo de Entomología, Universidad del Valle, Santiago de Cali, Colombia (MUSENUV) and the US National Mosquito Collection, Smithsonian Institution, Washington, DC, USA (USNMC), and original descriptions, keys, summaries and revisions from the published literature were examined. Primary publications for identification of Neotropical *Anopheles* include: key to genus *Anopheles* based on the morphology of male genitalia (Amazonian Brazil) [39]; keys to genus *Anopheles* based on the morphology of male genitalia, females and larvae (Venezuela) [40–44]; keys to *Anopheles* females and larvae (Brazil and other countries of South America) [45, 46]; key to *Anopheles* subgenus *Nyssorhynchus* Blanchard, 1902 females (western Venezuela) [47]; keys to *Anopheles* subgenus *Nyssorhynchus* based on the morphology of male genitalia, females and larvae (Amazonian Region) [48]; revision of all stages of *Anopheles* subgenus *Nyssorhynchus*, Albimanus Section [49]; genus *Anopheles*, comprehensive (North and South America) [32, 50]; keys to genus *Anopheles* females and larvae (Colombia) [51]; keys to genus *Anopheles* females and larvae (Venezuela, eastern and western South America, Central America and Panama) [52–55]; key to *Anopheles* subgenus *Kerteszia* Theobald, 1905 females [56]; keys to genus *Anopheles* male genitalia, larvae and eggs (Neotropics) [57]; revision of all stages of *Anopheles* subgenus *Nyssorhynchus*, Argyritarsis Section [58]; key to larvae (Venezuela) [59]; summary of *Anopheles* subgenus *Nyssorhynchus*, with definition of the Myzorhynchella Series [30]; key to females of *Anopheles* subgenus *Nyssorhynchus* (Venezuela) [60]; key to *Anopheles* females (Central America) [61]; key to *Anopheles* females (in Spanish) (Central America) [62]; and, revision of *Anopheles* subgenus *Kerteszia* [63]. From these available published literature records, we initiated the keys using [32, 49, 50, 56, 63] and [61].

Based on the published literature and specimens of additional species deposited in the previously mentioned collections [58–60], we present a list of species included in the identification keys. Table 1 shows the traditional classification of the genus *Anopheles*, including formal and informal groups, species authorship, and the date of publication.

The subgenus *Nyssorhynchus* includes the Albimanus [49], Argyritarsis [58], and Myzorhynchella Sections [30, 64]. The Albimanus and Argyritarsis Sections further include series, groups, subgroups, and species complexes [65–81]. The classification into section categories does not indicate monophyletic groups as shown in phylogenetic studies using morphology [25] and sequence data [26, 27]. Currently, the internal classification of *Nyssorhynchus* is primarily based on morphological similarities of the adults, males, and females, and it is herein adopted for convenience (Table 3). Species complex, such as *An. arthuri* Unti, 1941, includes additional three phylogenetic taxa, which were found in studies using mitochondrial and nuclear genes [69, 81]. Despite these taxa have not been formally described, they are included in Table 2, and details for species identification are provided in Sallum et al. [33, 34].

**Table 3:** Valid species of the subgenus *Nyssorhynchus* of *Anopheles* found in South America, grouped by series and informal groupings

Series	Group	Subgroup	Complex	Species, authorship, date
Albimanus [20]				*albimanus* Wiedemann, 1820^b^
Oswaldoi [20]	Oswaldoi [20]	Oswaldoi [20]		*aquasalis* Curry, 1932^a^
				*evansae* (Brèthes, 1926)^a^
				*galvaoi* Causey, Deane & Deane, 1943^a^
				*ininii* Senevet & Abonnenc, 1938
				*konderi* Galvão & Damasceno, 1942 (*s.s.*)^a^
				*oswaldoi* (Peryassú, 1922) (*s.s.*)^a^
				*rangeli* Gabaldon, Cova Garcia & López, 1940^a^
				*sanctielii* Senevet & Abonnenc, 1938
				*trinkae* Faran, 1979
			Nuneztovari [65, 66]	*dunhami* Causey, 1945^a^
				*goeldii* Rozeboom & Gabaldon, 1941^a^
				*nuneztovari* Gabaldon, 1940 (*s.s.*)^a^
		Strodei [20]		*albertoi* Unti, 1941^a^
				*arthuri* Unti, 1941 (*s.s.*)^a^
				*rondoni* (Neiva & Pinto, 1922)^a^
				*striatus* Sant’Ana & Sallum, 2016^a^
				*strodei* Root, 1926^a^
			Benarrochi [67]	*benarrochi* Gabaldon, Cova Garcia & López, 1941 (*s.s.*)^a^
		Triannulatus [20]		*halophylus* Silva-do-Nascimento & Lourenço-de-Oliveira, 2002^a^
				*triannulatus* (Neiva & Pinto, 1922) (*s.s.*)^a^
Albitarsis [30]	Albitarsis [30]		Albitarsis [68]	*albitarsis* Lynch Arribálzaga, 1878 (*s.s.*)^a^
				*deaneorum* Rosa-Freitas, 1989^a^
				*janconnae* Wilkerson & Sallum, 2009^a^
				*marajoara* Galvão & Damasceno, 1942^a^
				*oryzalimnetes* Wilkerson & Motoki, 2009^a^
	Braziliensis [30]			*braziliensis* (Chagas, 1907)^a^
Argyritarsis	Argyritarsis [30]			*argyritarsis* Robineau-Desvoidy, 1827^a^
				*sawyeri* Causey, Deane, Deane & Sampaio, 1943^a^
	Darlingi [30]			*darlingi* Root, 1926^a^
	Lanei [30]			*lanei* Galvão & Franco do Amaral, 1938^a^
	Pictipennis [30]			*atacamensis* González & Sallum, 2010^a^
				*pictipennis* (Philippi, 1865)^a^
Myzorhynchella				*antunesi* Galvão & Franco do Amaral, 1940^a^
				*guarani* Shannon, 1928^a^
				*lutzii* Cruz, 1901 (*s.s.*)
				*nigritarsis* (Chagas, 1907)^a^
				*parvus* (Chagas, 1907)^a^
				*pristinus* Nagaki & Sallum, 2010^a^

*Note*: Sources of fourth-instar larvae and male genitalia photographed for the illustrations are denoted as: ^a^ FSP-USP, Brazil; ^b^ MUSNUVE

## Conclusion

Taxonomic information and identification keys for species of South American *Anopheles* were updated and revealed the need for further morphology-based studies and descriptions of species of several complexes, species which have been defined on the basis of DNA sequence data but have not been formally named.

## Data Availability

Not applicable.
